# Genome-wide analysis of microRNAs identifies the lipid metabolism pathway to be a defining factor in adipose tissue from different sheep

**DOI:** 10.1038/srep18470

**Published:** 2015-12-22

**Authors:** Xiangyang Miao, Qingmiao Luo, Xiaoyu Qin, Yuntao Guo

**Affiliations:** 1 Institute of Animal Sciences, Chinese Academy of Agricultural Sciences, Beijing, 100193, China

## Abstract

MicroRNAs are short (17–24 nt) non-coding RNAs that are involved in post-transcriptional regulation of gene expression in multicellular organisms by affecting both the stability and translation of mRNAs. In recent years, deep sequencing of the transcriptome is increasingly being utilized with the promise of higher sensitivity for the identification of differential expression patterns as well as the opportunity to discover new transcripts, including new alternative isoforms and miRNAs. Here, we utilized RNA-seq technology to perform a genome-wide analysis of miRNAs from the adipose tissue of the two species of sheep to look for clues that might explain the fat deposition differences between the sheep. The RNA-seq analysis detected 3132 miRNAs from the adipose tissue of the Small-tail Han and Dorset sheep, of which 2893 were defined as potential new miRNAs. In addition, 54 miRNAs were differentially expressed between the two breeds of sheep. Gene ontology and pathway analyses of the predicted target genes that negatively associated with the differentially expressed miRNAs revealed that there was less active lipid metabolism in the adipose tissue of Small Tail Han sheep. This study can help understand the underling mechanisms responsible for the morphological differences related to fat deposition between two breeds of sheep.

A microRNA (miRNA) is a small non-coding RNA molecule (about 17–24 nucleotides in length) found in plants, animals, and some viruses, which functions in transcriptional and post-transcriptional gene expression regulation by affecting both the stability and the translation of messenger RNAs (mRNA)^1^,^2^. miRNAs are transcribed by RNA polymerase II as part of capped and polyadenylated primary transcripts (pri-miRNAs) that can be either protein-coding or non-coding. The primary transcript is cleaved by the Drosha ribonuclease III to produce a stem-loop precursor miRNA (pre-miRNA) of approximately 70-nt, which is further cleaved by the cytoplasmic Dicer ribonuclease to generate a mature miRNA and an antisense miRNA star (miRNA*) product. The mature miRNA is incorporated into an RNA-induced silencing complex (RISC), which recognizes the target mRNAs through imperfect base pairing with the miRNA and most commonly results in translational inhibition or destabilization of the target mRNA^3^. Increasing evidence shows that miRNAs play important roles in multiple biological and metabolic processes, including cell development and differentiation, signal transduction, and diseases^4^,^5^,^6^.

In recent years, there has been an increase in utilization of deep sequencing of the transcriptome for the identification of differential expression as well as for the opportunity to discover novel transcripts, including new alternative isoforms and miRNAs^7^,^8^,^9^,^10^. In the present study, the fat tissues from two distinctive breeds of livestock sheep (Small Tail Han and Dorset) were profiled by small RNA deep sequencing technology. Dorset sheep originated in England and have a medium body size with good muscle conformation to produce a desirable carcass, while the Small Tail Han sheep (called “Han sheep” hereafter) are excellent local breeds in China that provide good flavored meat and are plentiful in fat. In contrast to the Dorset sheep, which have rapid growth, the Han sheep have high prolificacy with a mean litter size of 2.61^11^, compared to 1.45 for Dorset^12^. Significant differences in the fat deposition between these two breeds have spurred increasing interest in the characterization of the genetic profiles of these sheep^13^,^14^,^15^,^16^. Fat deposition is a major factor that affects the quality of the meat. For a variety of livestock, there are important differences between species that can only be partially explained by digestive process differences^13^. This suggests that the factors and mechanisms responsible for the differences in fat deposition, which have not been elucidated completely, could aid in the development of new strategies to modulate fat deposition and improve the nutritional value of meat. Thus, the present study aims to examine differentially expressed miRNAs from two species of sheep in order to identify key pathways that might be related to the mechanism of fat deposition using bioinformatics.

## Results

### Summary of the raw sequence reads

**Figure 1:**
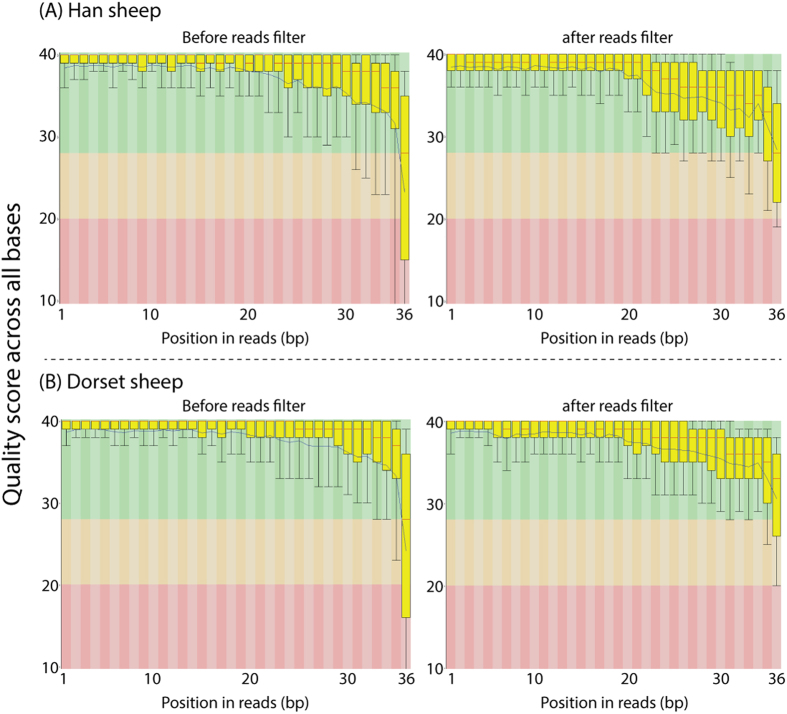
Read quality diagrams before and after quality filtering in the Han (A) and Dorset (B) sheep. These data indicate that high-quality sequencing reads were retained in downstream analyses.

In the present study, miRNA from the fat tissue of Han and Dorset sheep were sequenced. With a quality filter at Q-score >30 (Fig. 1), we obtained 7.78 and 4.19 million raw sequence reads per sample from the Han and Dorset libraries, respectively (Table 1). Of these reads, 79.35% (5.80/7.78 million reads, Han) and 56.75% (2.11/4.19 million reads, Dorset) were mapped to the ovine genome Oar_v3.1. The difference in the mapping rate between the Han and Dorset sheep libraries was not certain but could be a reflection of the fundamental genome difference between the two distinctive sheep breeds. As shown in Table 1, about 2% of the total reads (Han 2.4% and Dorset 1.8%) were annotated with well-known miRNAs for sheep, cow, pig, and human in the Sanger miRBase database, whereas 74.6% (Han) and 50.4% (Dorset) of total reads were identified as new miRNAs that were mapped to the ovine genome but not reported yet in the Sanger miRBase database. To explore these further, additional new miRNA prediction procedures were applied on these reads to identify reliable and unique new miRNA species and the results are described in the next section.

### Prediction of new miRNAs

**Figure 2:**
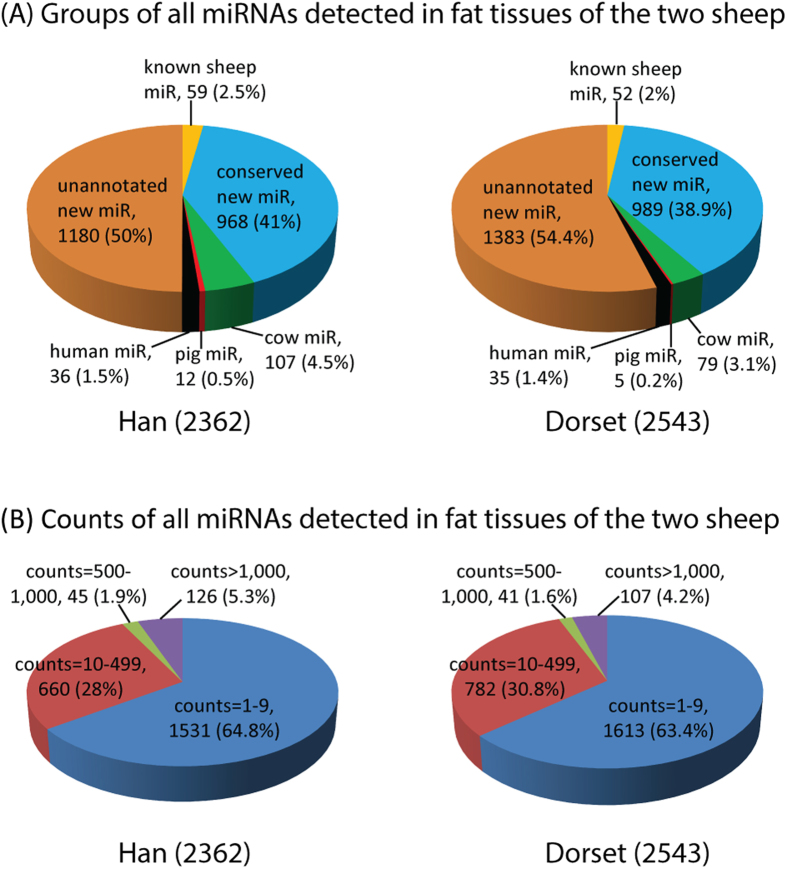
The union 3132 miRNAs detected between the two libraries of ovine fat tissues.

In the present study, we first mapped all of the sequencing reads to the ovine miRNAs in the Sanger miRBase database, and then the remaining reads were subjected to a hairpin based miRNA prediction process described in the Methods section to ensure reliable miRNA identification. The reads with predicted hairpin structures were mapped to other species (human, cow, and pig) conserved miRNAs in the Sanger miRBase database, and the remaining unmapped but hairpin-structured reads were considered potential new miRNAs without known miRNA annotations. Thus, we identified three groups of miRNAs: (1) miRNAs previously described in sheep, (2) miRNAs described in species other than sheep and (3) new miRNAs without any known annotations. In total, 3132 miRNAs were identified (coming from the union of 2362 in the Han and 2543 in the Dorset) with 1 or more read in the two small RNA libraries of the ovine fat tissues. Of the total miRNA identified 92%, (2893, which came from the union of 2148 in the Han and 2372 in the Dorset) were predicted to be new miRNAs that were not reported in the current sheep miRBase database (Fig. 2A). Forty-one percent of the new miRNAs (1197 of 2893, which was from the union of 968 Han and 989 Dorset) were conserved miRNAs from other species (human, cow and pig), and the other 59% of the new miRNAs (1696 of 2893, from the union of 1180 Han and 1383 Dorset) were unannotated new miRNAs (Fig. 2A and Supplementary Table S1).

### Differential expression

**Figure 3:**
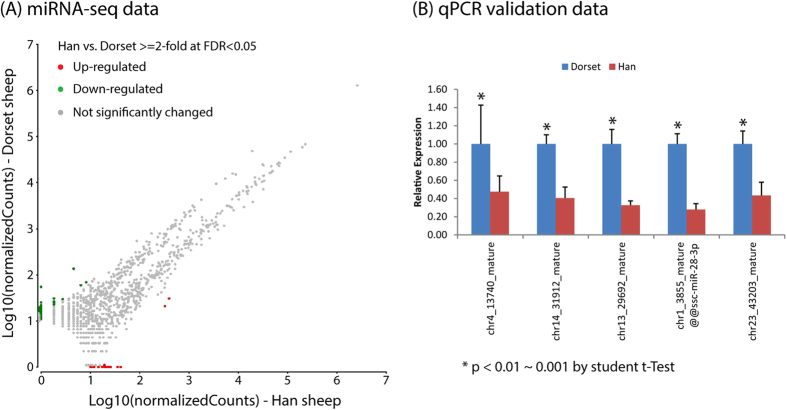
(**A**) Scatter plot of the library size normalized sequencing read counts data from the two samples showing statistically differentially expressed miRNAs; (**B**) qPCR validation of 5 miRNAs.

We detected 3132 miRNAs with 1 or more sequencing read in the two libraries from the ovine fat tissues, of which ~36% were miRNAs with at least 10 reads and ~64% were miRNAs with 1–9 reads (Fig. 2B). In order to minimize the sequencing data noise in the statistical analysis, we focused on the 1234 miRNAs that had at least 10 counts in the two samples. Using the R package “EBSeq”, we identified 54 (35 down and 19 up) differentially expressed miRNAs (4.4% of the 1234 miRNAs) at FDR adjusted p-value < 0.05 and absolute value of log2Ratio> = 1 (Han vs. Dorset) (Supplementary Table S2). Of the 54 differential miRNAs, 52 were new miRNAs and 2 were known cow miRNAs. The normalized count data are displayed in Fig. 3A, showing the statistically significant differentially expressed miRNAs.

### Target prediction of the differential miRNAs

We used the MiRanda program to predict the potential targets of those differentially expressed miRNAs, and 7,510 genes were predicated to be potential targets of the 54 differentially expressed miRNAs in the Han and Dorset sheep. When examining the relationship between the miRNAs and the targets, only those miRNA-target pairs with opposite up- or down-regulations are biologically meaningful. Thus, we integrated the expression profiles of the miRNAs with mRNA data we previously obtained in a separate study^17^ to predict the miRNA-mRNA interactions. The mRNA data was obtained to characterize the gene expression profiles in these sheep breeds, and we took advantage of RNA sequencing technology with the aim of identifying important genes regulating the metabolisms in adipose tissue of the two distinct breeds of sheep. We obtained high quality sequencing reads, 85.9% (47.39/55.17 million reads, Han) and 86.1% (54.83/63.65 million reads, Dorset) that were uniquely aligned to the Oar_v3.1 sheep reference genome, and over 76% of the bases in the mapped reads corresponded to mRNA (Han: 76.3%; Dorset: 78.1%). Using the R package “EBSeq”, we identified 602 differentially expressed genes at FDR <0.05 and fold-change > = 2 (Han vs. Dorset sheep). Using the 602 genes, gene ontology analysis showed that 30 out of 56 (53.6%) of the significantly enriched biological processes (p < 0.01 and FDR<0.2) were metabolism related, of which the most significant one was “triglyceride biosynthetic process”. The KEGG pathway analysis indicated the down-regulation of several fat metabolic pathways as well. Thus, we utilized mRNA data from our previous study^17^, which was conducted on the same set of animals, and compared it with the current miRNA data in order to identify potential targets of the miRNAs. Only the negatively associated miRNA-mRNA pairs were considered a potential miRNA-mRNA interaction (|log2(ratio)|> = 1, p < 0.05). We found 125 genes were predicated as potential targets of the 47 differential miRNAs (Supplementary Table S3).

### Annotations with gene ontology (GO) and KEGG pathways

**Figure 4:**
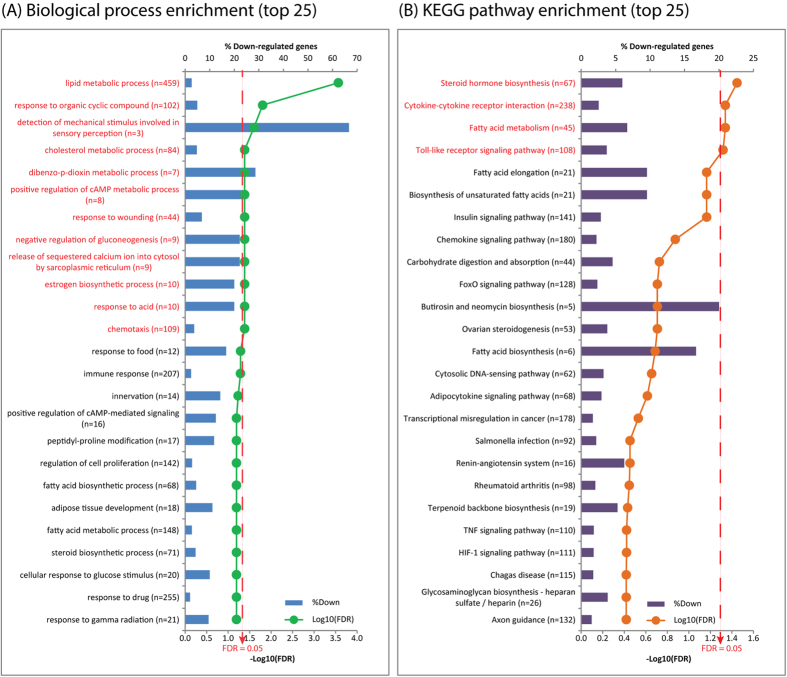
Gene Ontology (A) and KEGG pathway (B) enrichment analysis were performed using 125 differential target genes that were negatively associated with 47 differential miRNAs. Titles in red indicate significantly enriched biological processes and KEGG pathways.

Among the 125 potential target genes, the GO analysis indicated that the down-regulated target genes in miRNA sample of the Han sheep were significantly overrepresented in 12 biological processes at p < 0.01 and a FDR < 0.05, of which the lipid metabolic process was the most significant term with 12 down-regulated target genes enriched, showing fold changes bewteen −2.3 and −88.4 (Fig. 4A and Table 2). None of the up-regulated target genes showed statistically significantly enriched biological processes at a FDR < 0.05. In order to illustrate the regulatory network between the differentially expressed miRNAs and their target genes, we generated a miRNA-mRNA interaction network for the 47 miRNAs and 125 target genes (Supplementary Figure S1). The pathway analysis revealed that the down-regulated target genes in the miRNA sample of the Han sheep were significantly overrepresented in 4 pathways at p < 0.01 and a FDR < 0.05, including the fatty acid metabolism pathway, which was made of 3 down-regulated target genes with fold changes ranging between −2.4 and −6.3-fold (Fig. 4B). There were no statistically significantly enriched biological pathways at a FDR < 0.05 for the up-regulated target genes. Moreover, the 12 down-regulated target genes that were enriched in the lipid metabolic process were negatively regulated by 10 miRNAs, including 1 known cow miRNA, 4 human and pig conserved new miRNAs, and 5 unannotated new miRNAs (Table 2).

### Validation of differential miRNA expression

Five differential miRNAs were selected from the 47 miRNAs that were negatively correlated with their target genes for the stem-loop qPCR validation. The direction of the fold-changes detected by qPCR was similar to those observed the miRNA-seq analysis (Fig. 3A,B). These consistencies validate the miRNA-seq data. In addition, we examined the mRNA expression of the targets of these 5 miRNAs by performing qPCR. All 5 of the gene targets were up-regulated in the Dorset sheep, which corresponds with the fact that their corresponding miRNAs were up-regulated in the Han sheep (Table S4).

## Discussion

Technological advancements have resulted in an increased discovery of new miRNAs. Using RNA deep sequencing technology, we discovered a large number of new miRNAs in the two different breeds of livestock sheep. These new miRNAs were all confirmed as having a hairpin secondary structure in their predicted precursor sequences. However, several recent studies have reported identifying only a small number of new miRNAs in cattle (36 novel miRs)^18^, pig (61 novel miRs)^19^, goat (35 novel miRs)^9^ and horse (329 novel miRs)^10^ using the same technology. The difference between the previous studies and ours is that these authors used relatively more restricted prescreening procedures to rule out sequences that were detected at a low count level (e.g. <10 reads in a single sample) or a low sample frequency (e.g. in <50% of samples). While, our study aimed to comprehensively identify new species of miRNAs with as few as 1 sequence count in any sample, as long as the sequence reads had high quality Q-scores and mapped to the sheep genome, and the sequences had a confirmed secondary structure in the predicted miRNA precursor. In our study, we believe that the high quality sequencing data ensured the reliable discovery of new miRNAs in these breeds of sheep, and the confirmation of secondary structures rendered a meaningful identification of the new miRNAs. In the Rfam database (August 2012, release 11.0), 2208 RNA families have been defined so far (http://rfam.xfam.org/), and there are a total of 28645 miRNA entries for all species in the latest miRBase database (release 21 in June 2014 at http://www.mirbase.org/), of which 2588, 793, 411 and 153 are for known human, cow, pig, and ovine mature miRNAs, respectively. In the present study, the known ovine miRNAs accounted for only 0.57% (Han) and 0.67% (Dorset) of all the miRNAs identified in the two libraries, whereas over 90% of all miRNAs were new miRNAs, either conserved miRNAs of other species or new miRNAs without any known miRNA annotation in the Sanger miRBase database. Further studies of these new miRNAs may unveil their biological significance in the regulation of fat metabolism in the adipose tissues of the livestock sheep.

It is estimated that up to 30% of genes might be regulated by miRNAs^20^. In a separate study, we have demonstrated a predominant down-regulation of massive lipid metabolic processes in the adipose tissue of Han sheep. However, the regulatory mechanisms for the down-regulation were not fully elucidated. The current study has identified 54 differentially expressed miRNAs in the two breeds of the livestock sheep (Han and Dorset), of which 35 were down-regulated and 19 were up-regulated in the Han sheep. The target prediction and integration of these differentially expressed miRNAs with mRNA-seq data generated in that separate study helped to identify the most significant miRNAs and the potential target genes that could be responsible for the differential regulation of lipid metabolism in the adipose tissue of the two breeds. Through those procedures, the current study identified 12 down-regulated target genes that were negatively correlated with 10 up-regulated miRNAs in the Han sheep, and all 12 of genes were enriched in the lipid metabolic process. Among the target genes, we can examine their functional roles to see how potential mechanisms might be uncovered by examining these 12 genes together. Three of the genes are known key regulators of adipocytes. AACS is an enzyme that utilizes ketones in lipogenesis, which is crucial for adipocyte differentiation^21^, THRSP is expressed in adipocytes and is suggested to play a role in regulating lipid metabolism^22^, and LEP is secreted by adipocytes and plays a role in the regulation of body weight^23^. Two of the genes, ACACA and INSIG1 are insulin sensitive. ACACA is a flux determining enzyme of the lipogenic pathway that coordinates fatty acid synthesis in response to insulin^24^ and INSIG1 is insulin induced and regulates the cholesterol content of cells^25^. In addition, ELOVL6 and HSD17B12 are both elongases that are responsible for controlling the overall balance of fatty acid composition^26^,^27^, and MOGAT2 catalyzes the synthesis of triacylglycerol, which is a step required for dietary fat absorption^28^. CYP11A1 encodes a member of the cytochrome P450 superfamily and is involved in the synthesis of lipids in the mitochondria^29^. HPGD is responsible for the metabolism of prostaglandins and is known to function in a variety of physiological processes^30^. IDI1 is important in cholesterol synthesis^31^, and GDE1 is involved in fatty acid metabolism^32^. These 12 genes, can be used to identify key components in the lipogenesis pathway that might provide clues for defining key difference between these 2 breeds of sheep.

Intriguingly, 9 of the 10 up-regulated miRNAs were newly identified in the current study, implicating the importance of our comprehensive approach for the discovery of novel miRNAs. In general, our current study revealed a less active lipid metabolism of the adipose tissue of Han sheep as compared with Dorset sheep. This may suggest an inhibitory feedback mechanism that is similar to what a recent study demonstrated. These researchers found that in the adipose tissue of obese diabetic individuals there is a down-regulation of the acetyl-CoA metabolic network, which is recovered after weight loss^33^. Such an inhibitory feedback mechanism might originate from more fat deposition due to an up-regulation of miRNAs and down-regulation of certain lipid metabolism associated genes, and the accumulation of some metabolites might ultimately alter the whole process of lipid metabolism.

The data described here utilized RNA-Seq technology, which, in general, has enabled us to generate an unprecedented global view of the transcriptome and its organization for a number of species and cell types. Therefore, RNA-Seq has the potential to provide more accurate and comprehensive information, particularly for the identification of novel molecules. A major limitation of RNA-Seq technology is the bioinformatic challenge that comes with it, which has to be overcome in order to reduce errors in image analysis and base calling and to also remove low-quality reads. We identified a number candidate miRNAs and also their corresponding targets, these data provide a starting place for further studies that are still needed to verify the physiological functions of the candidate genes. In addition, our study was simply looking for differences in the fat distributions between the breeds of sheep, but we also have the potential to determine if there are differences between males and female in each breed, which could not be analyzed in our study because all of samples were pooled for the analysis.

## Conclusions

Our genome-wide miRNA expression profiling study identified 2893 new miRNAs with the aid of various bioinformatics tools, Among these miRNAs, 1197 were conserved miRNAs of other species (human, cow, and pig), and 1696 were completely new miRNAs that are not reported anywhere and require further experimental validation. Differential expression was statistically observed in 54 miRNAs in the adipose tissue of the Han sheep compared with the Dorset sheep. Gene ontology and pathway analysis of the predicted target genes that negatively associated with the differential miRNAs demonstrated less active lipid metabolism in the adipose tissue of the Han sheep. These data serve as a unique resource that can be used to address important issues related to understanding the mechanisms responsible for the morphological differences between two distinctive breeds of livestock sheep.

## Methods

### Animals and preparation of fat tissues

All of the procedures involving animals were approved by the animal care and use committee at Institute of Animal Sciences, Chinese Academy of Agricultural Sciences where the experiment was conducted. All of the experiments were performed in accordance with the relevant guidelines and regulations set by the Ministry of Agriculture of the People’s Republic of China. Healthy 2-year-old ewes were obtained from the Qingdao Aote Sheep Farm (Shandong, China) and two different breeds were analyzed in this study: Dorset and Han sheep. All of the animals were raised under the same conditions of free access to water and food in natural lighting. The body weight, carcass weight, growth rate and adipocyte volume (μm^3^) was 75.3 kg, 33.8 kg, 11.41%, 1.71 kg/d and 167.8, respectively for the Han sheep and was 80.5 kg, 37.8 kg, 12.16%, 1.82 kg/d and 150.3, respectively for the Dorset sheep. Table S5 shows a comparison of the fatty acid composition between the 2 breeds of sheep. Six sheep from each breed (three were males and three were females) were selected randomly, but were as close as possible to the median average daily weight and carcass weight of their group. The amount of fat in the male animals was significantly lower than that of the females, which is consistent with the literature^34^. Adipose tissue samples from the backfat of the sheep were collected within 30 min after slaughter, cut into pieces of 8 mm^3^ and quickly placed into an RNA preservation solution (RNA-later, Life Technologies, Carlsbad, CA) for 24 hours at room temperature, and then the tissue blocks were transferred to a −80 °C freezer for long-term preservation.

### Library preparation and miRNA sequencing

The fat tissue was disrupted with liquid nitrogen and the total RNA was extracted using Trizol reagent (Cat.#15596026, Life Technologies, Carlsbad, CA), according to the manufacturer’s protocol. The quality and quantity of the RNA samples were assessed on a Bioanalyzer 2100 system using an RNA 6000 Nano kit (Agilent Technologies, Palo Alto, CA). After the total RNAs were extracted, two small RNA libraries were prepared as described in the Illumina® TruSeq™ Small RNA Sample Preparation protocol. The RNA from the 6 sheep in each group was pooled to generate the two libraries^35^. Firstly, the 3′ adaptor and 5′ adaptor were ligated to the RNA by T4 RNA ligase, and then reverse transcription was carried out with SuperScriptII reverse transcriptase (Invitrogen) to generate cDNA. The resulting cDNAs were then amplified to generate the small RNA library^36^. DNA size and the purity of the cDNA library was checked using a high sensitivity DNA 1000 kit on a Bioanalyzer 2100 system (Agilent Technologies, Santa Clara, CA), and quantification of the cDNA libraries was performed with a Qubit™ dsDNA HS kit on a Qubit® 2.0 Fluorometer (Life Technologies, Carlsbad, CA). The cDNA libraries were then subjected to single-end sequencing on the Illumina Genome AnalyzerIIx(GAIIx) using the proprietary Solexa sequencing-by-synthesis method at the Shanghai Biotechnology Corporation (Shanghai, China), according to the manufacturer’s recommended cycling parameters. Image analysis and base calling was performed with the Illumina built-in SCS2.8/RTA1.8 software.

### Reads mapping and annotation

To obtain clean and unique small RNA reads, the fastx_toolkit (v0.0.13.2, downloadable at http://hannonlab.cshl.edu/fastx_toolkit/) was used to filter off (1) low quality reads, (2) reads with 5′ primer contaminants, (3) reads without a 3′ primer, (4) reads without the insert tag, (5) reads with poly (A) or simple repeats, and (6) reads shorter than 18 nt. The clean reads were screened against and mapped to the latest ovine genome assembly (Oar_v3.1, released Sept. 20, 2012) using the SOAP program (SOAP aligner v2.21, http://soap.genomics.org.cn/)^37^. After mapping, we used the CLC Genomics Workbench 5.5 tool to blast the clean reads against the Rfam database (http://www.sanger.ac.uk/software/Rfam) and the GenBank noncoding RNA database (http://blast.ncbi.nlm.nih.gov/) to annotate and remove reads for mRNA, rRNA, tRNA, snRNA and snoRNA. The remaining sequences were then searched against the mature miRNAs of human and animals including cow, pig, and sheep in miRBase (Release 20)^36^ to identify known conserved miRNA homologs in the livestock sheep. Only those small RNAs whose mature and precursor sequences were perfectly matched to known ovine miRNAs in the miRBase were considered conserved miRNAs. Sequences that were identical to or related (i.e. no more than one mismatch in the seed sequence and a few end nucleotides fluctuation in the entire length) to the reference mature miRNAs, were annotated as miRNA candidates. The miRNA candidates were then clustered into categories according to sequence similarity, and the sequences varying only in length and/or a few end nucleotides were gathered under the same miRNA identifier.

### Identification of conserved and new miRNAs

In order to discover potential new miRNAs, the surrounding 300 bases (150 upstream and downstream) flanking each unique miRNA candidate sequence were obtained and their folding secondary structures were determined using the miRDeep program^38^. If a hairpin structure with a free energy of hybridization lower than −20 kcal/mol was predicted, the RNA sequence was subjected to the miRDeep analysis, which predicts whether the input RNA sequence is a genuine pre-miRNA-like hairpin sequence. After prediction, the resulting potential miRNA loci were examined carefully based on the distribution and numbers of small RNAs on the entire precursor regions. Those sequences residing in the stem region of the stem-loop structure and ranging between 20–22 nt with free energy hybridization lower than −20 kcal/mol were considered as potential new miRNAs^39^.

### Determination of miRNA differential expression

The differentially expressed miRNAs were identified by the R package “EBSeq” program using raw small RNA counts as input, and quantile normalization was applied for variable library sizes^40^. EBSeq is an empirical Bayesian approach that models a number of features observed in the RNA-seq data. A list of the differentially expressed genes with a 2-fold difference was generated and controlled with a false discovery rate (FDR) at 0.05 in an experiment comparing two biological conditions without replicates. When replicates are not available, EBSeq estimates the variance by pooling similar genes into a certain number of bins, and this approach works well when there are no more than 50% differentially expressed genes in the data set^40^.

### Collection and analysis of the mRNA data used for prediction of the miRNA targets

All of the mRNA data was obtained from fat collected from the same animals that were used for the miRNA analysis at the same relative time (age) and using a similar procedure. The details of these experiments can be found in our previous publication^17^.

### Target prediction of differentially expressed miRNAs

To identify potential miRNA target genes, the differentially expressed miRNAs were analyzed using the miRanda program (downloadable at http://www.microrna.org/microrna/getDownloads.do) in which the seed sequence at miRNA 2–8 bp was mapped against the 3’ UTR regions of known mRNA genes. Next, by integrating the mRNA and miRNA data, only those mRNAs that were negatively correlated with the specific miRNA were considered as targets for further analysis.

### Annotations of negatively associated genes with differentially expressed miRNAs

In order to extract biological significance from the potential target genes associated with the differentially expressed miRNAs, we conducted gene enrichment analyses using the Gene Ontology (GO)^41^ and Kyoto Encyclopedia of Genes and Genomes databases (KEGG, http://www.genome.jp/kegg)^42^,^43^.

### Quantitative PCR (qPCR)

Three replicates per breed were used for the qPCR analysis. Firstly, reverse transcription was carried out on 1 μg RNA using the One-Step miRNA RT Kit (Cat#:33-30120, SinoGene, Beijing China), and then the synthesized cDNAs were used as templates in the stem-loop qPCR^44^. SYBR Green chemistry was utilized to measure the gene expression level, and the qPCR reactions were performed in the StepOnePLUS system (Applied Biosystems Inc.), with a 20-μl reaction consisting of 10 ng of cDNA, 200 uM of forward and reverse primers each, and 2 μl of 2x SYBR Green qPCR Mix (with ROX, Cat#: 22-10102, SinoGene Beijing, China). The qPCR program was 95 °C for 15 min, followed by 45 cycles of 95 °C for 15 sec, 60 °C for 15 sec and 72 °C for 45 sec, and a final stage of dissociation analysis. The comparative Ct method and internal control miRNA gene U6 were used for the calculations of the relative expression levels of the genes. All of the qPCR reactions yielded a single peak on the dissociation curve, indicating specific amplifications.

### Statistical Analyses

All of the data are presented as the means ± the SD. When comparisons were made, a Student’s t-test was performed and p < 0.05 was considered statistically significant.

## Additional Information

**How to cite this article**: Miao, X. *et al.* Genome-wide analysis of microRNAs identifies the lipid metabolism pathway to be a defining factor in adipose tissue from different sheep. *Sci. Rep.* **5**, 18470; doi: 10.1038/srep18470 (2015).

## Supplementary Material

Supplementary Information

Supplementary Table S1

Supplementary Table S2

Supplementary Table S3

Supplementary Table S4

Supplementary Table S5

## Figures and Tables

**Table 1:** miRNA mapping statistics.

Categories	Dorset	HanSheep
sheep_MiRNA	28018 (0.67%)	44593 (0.57%)
newMiRNA_Annotated	2113853 (50.37%)	5803535 (74.60%)
newMiRNA_UnAnnotated	193153 (4.60%)	187372 (2.41%)
cow_miRNA	45632 (1.09%)	136101 (1.75%)
pig_miRNA	471 (0.01%)	1060 (0.01%)
human_miRNA	543 (0.01%)	1225 (0.02%)
unmapped	1815036 (43.25%)	1606389 (20.65%)
total	4196706	7780275

**Table 2:** 12 down-regulated genes in Han vs. Dorset sheep that are negatively associated with 10 miRNAs and enriched in lipid metabolic process.

miRNA.ID	Log 2 Ratio (Han/Dorset, miRNA)	FDR (miRNA)	Target_ID	Log 2 Ratio (Han/Dorset, mRNA)	FDR (mRNA)
bta-miR-18a	4.12	4.71E-02	CYP11A1	−6.47	0
bta-miR-18a	4.12	4.71E-02	HPGD	−1.28	3.44E-06
chr2_7666_mature	9.97	4.80E-02	ELOVL6	−2.66	0
chr2_7666_mature	9.97	4.80E-02	THRSP	−1.84	0
chr2_8758_star	10.97	5.18E-03	AACS	−1.19	3.56E-08
chr3_10857_mature@@hsa-miR-29b-1-5p	10.91	6.13E-03	GDE1	−1.26	1.28E-09
chr3_10857_mature@@hsa-miR-29b-1-5p	10.91	6.13E-03	IDI1	−1.64	2.22E-16
chr3_10857_mature@@hsa-miR-29b-1-5p	10.91	6.13E-03	LEP	−2.15	0
chr6_17762_mature	3.68	8.10E-03	ACACA	−1.36	1.12E-11
chr7_20828_mature@@ssc-miR-22-3p	11.32	2.07E-03	ELOVL6	−2.66	0
chr7_20828_mature@@ssc-miR-22-3p	11.32	2.07E-03	INSIG1	−2.00	0
chr13_29469_mature@@hsa-miR-4749-5p	10.22	2.99E-02	ACACA	−1.36	1.12E-11
chr20_39415_mature@@ssc-miR-206	10.91	6.13E-03	LEP	−2.15	0
chr25_44658_mature	3.96	3.43E-03	ELOVL6	−2.66	0
chr26_45516_mature	10.10	3.77E-02	HSD17B12	−1.25	7.33E-10
chr26_45516_mature	10.10	3.77E-02	MOGAT2	−4.27	1.12E-07
