# Supplementary material for: Genome-wide analysis of microRNAs identifies the lipid metabolism pathway to be a defining factor in adipose tissue from different sheep
Source: Sci Rep. 2015 Dec 22;5:18470. doi: 10.1038/srep18470 (PMC4686875; doi:10.1038/srep18470)
Supplement: Supplementary Information [file srep18470-s1.doc]

Supplementary Information

**Genome-wide analysis of microRNAs identifies the lipid metabolism pathway to be a defining factor in adipose tissue from different sheep**

Xiangyang Miao^*^ Qingmiao Luo Xiaoyu Qin Yuntao Guo

Institute of Animal Sciences, Chinese Academy of Agricultural Sciences, Beijing, 100193, China

^*^Corresponding author: Xiangyang Miao; Tel: 86-10-62895663(China),

E-mail: [mxy32@sohu.com](mailto:mxy32@sohu.com)

Figure S1 miRNA-mRNA network for the different groups. Red indicates up-regulated target genes, Green indicates down-regulated target genes, pink indicates up-regulated miRNA, and blue indicates down-regulated miRNA.

Table S1 3132 detected miRNAs (groups and counts).

Table S2 1234 miRNAs with at least 10 counts in the two samples.

Table S3 125 target genes negatively associated with 47 miRNAs

Table S4 Validation of RNA-seq data by RT-PCR

Table S5 Comparison of the Fatty Acid Composition Between the 2 Breeds of Sheep


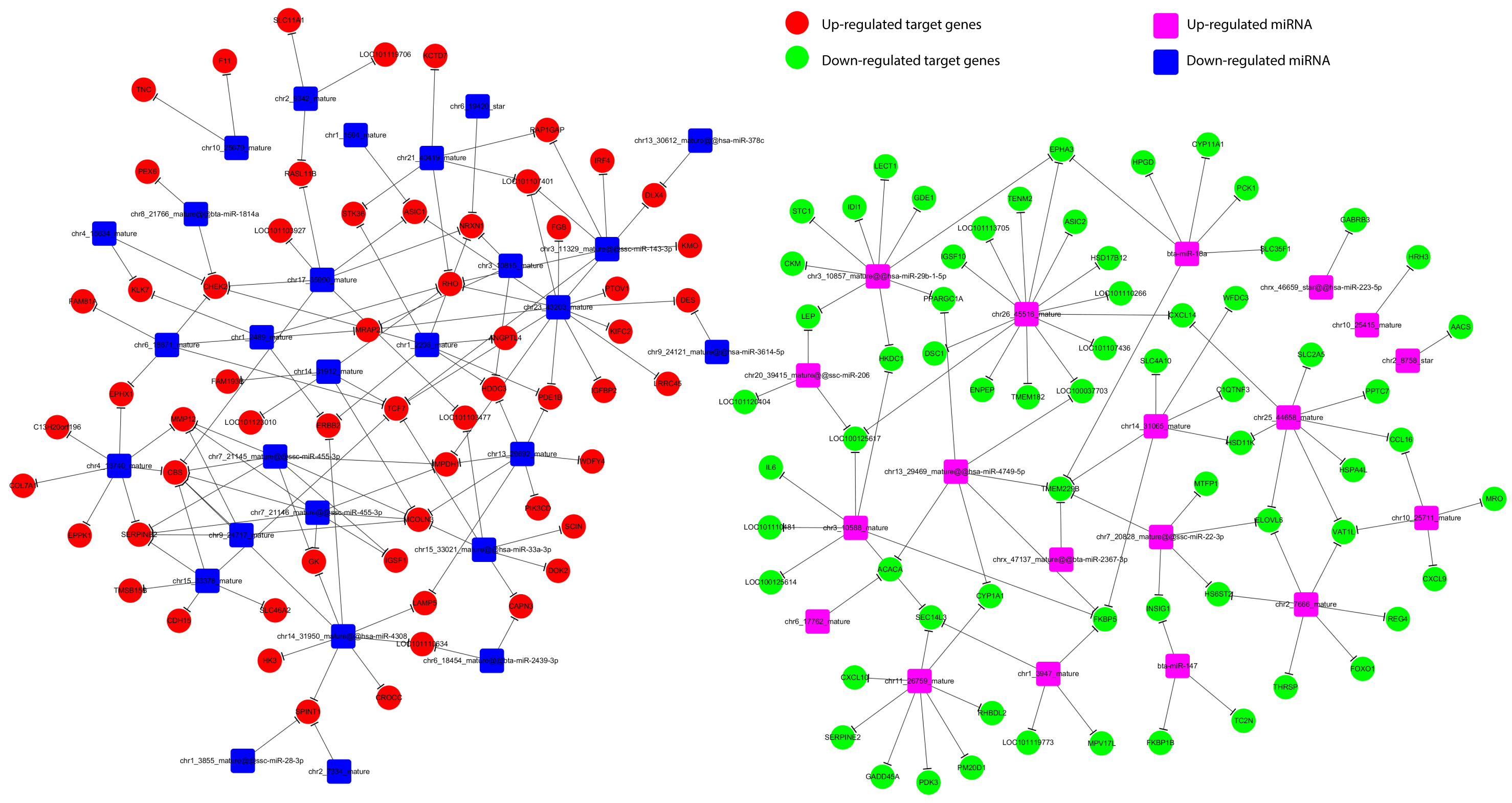


Figure S1 – miRNA-mRNA network
