# Supplementary material for: Genome-wide analysis of microRNAs identifies the lipid metabolism pathway to be a defining factor in adipose tissue from different sheep
Source: Sci Rep. 2015 Dec 22;5:18470. doi: 10.1038/srep18470 (PMC4686875; doi:10.1038/srep18470)
Supplement: Supplementary Table S5 [file srep18470-s6.doc]

Table S5. Comparison of the Fatty Acid Composition Between the 2 Breeds of Sheep

| Items | Small Tail Han Sheep | Dorset Sheep |
| --- | --- | --- |
| C16:0 | 230.15 | 275.38 |
| C16:1 | 125.72 | 158.57 |
| C18:0 | 227.39 | 247.53 |
| C18:1 | 170.23 | 61.70 |
| C18:1T | 139.41 | 183.43 |
| C18:2 (n-6) | 151.85 | 168.05 |
| C18:3 (n-3) | 103.50 | 82.71 |
| C18:3 (n-6) | 69.73 | 98.30 |
| C20:4 (n-6) | 116.41 | 191.31 |
| C20:5 (n-3) | 76.43 | 114.50 |
| C22:5 | 96.16 | 167.35 |
| C22:6 (n-3) | 63.14 | 95.75 |
| C24:0 | 59.70 | 94.80 |
| C24:1 | 67.50 | 93.25 |
| n-6/n-3 | 1.39 | 1.56 |
